# Health needs: the interface between the discourse of health professionals and victimized women^1^

**DOI:** 10.1590/0104-1169.3455.2555

**Published:** 2015-04-14

**Authors:** Rebeca Nunes Guedes de Oliveira, Rosa Maria Godoy Serpa da Fonseca

**Affiliations:** 2 Post-doctoral fellow, Escola de Enfermagem, Universidade de São Paulo, São Paulo, SP, Brazil. Scholarship holder from Fundação de Amparo à Pesquisa do Estado de São Paulo (FAPESP), Brazil, process 2013/06796-1; 3 PhD, Full Professor, Escola de Enfermagem, Universidade de São Paulo, São Paulo, SP, Brazil

**Keywords:** Violence Against Women, Family Heath Program, Needs Assessment

## Abstract

**Objective:**

to understand the limits and the evaluative possibilities of the Family Health Strategy regarding the recognition of the health needs of women who experience violence.

**Method:**

a study with a qualitative approach, grounded in the perspective of gender, and which adopted health needs as the analytical category. The data were collected through interviews with health professionals and women who made use of a health service, and were analyzed using the method of discourse analysis.

**Results:**

the meeting between the discourses of women who use the services and the professionals of the health service revealed, as the interface, human needs, as in the example of autonomy and of bonds. The understanding regarding the needs was limited to the recognition of health problems of physical and psychological natures, just as the predominance of the recognition of needs for maintaining life in the light of essentially human needs was revealed in the professionals' discourses as an important limitation of the practices.

**Conclusion:**

emphasis is placed on the perspective of gender as a tool which must be aggregated to the routine of the professional practices in health so as to confirm or deny the transformative character of the care in place regarding the recognition and confronting of the women's health needs.

## Introduction

In Brazil, the Family Health Strategy (ESF) has constituted the cornerstone for the viabilization of the Unified Health System (SUS), facilitating approximation between health professionals and the clientele in their defined areas of coverage, in this way revealing problems which had previously remained unknown by the services, as is the example with gender violence^(^ 1 ^)^. In the collective, the strategy represents the most fertile characteristic of the implementation of the practices in collective health in Brazil, being configured as the privileged locus for the recognition of health needs which, articulated with the other instances of the health network, seeks to meet these needs^(^ 2 ^)^.

In the ambit of women's healthcare, the ESF constitutes a space for implementing the National Policy for Integral Attention to Women's Health (PNAISM). Healthcare, taking into account gender inequalities, as well as the recognition and confronting of needs which can go beyond those of a biological and reproductive character, considering the specific social characteristcs, are principles which guide the current policy. However, the work processes which concretize these policies have been contradictory, as the practices end up being translated, on most occasions, into attending biological aspects of the female body^(^ 3 ^)^.

In relation to women's health, female vulnerability when faced with certain health issues is more related to the situation of discrimination in society than to biological factors. The historical and social construction of gender relationships has imputed, to women, poor and subordinate conditions of life, which are significant determinants of their health-illness process. The levels of poverty and life conditions in society have transformed over history, with a tendency which is unfavorable to women. This process is permeated by the undervaluing of female work, by the increase in the number of female heads of family, constituting the biggest number of poor families in society, by gender violence, and by the overload of the triple workday. These phenomena exemplify destructive processes in women's lives, which are closely related to their health needs^(^ 4 ^)^.

Gender violence, although characterized as a relational phenomenon between men and women, is principally imposed on women, constituting a health issue. It is estimated^(^ 5 ^)^ that this problem is a greater cause of deaths among women aged between 15 and 44 years old than cancer, malaria, traffic accidents and war. There is, furthermore, a Brazilian estimate that at least 35% of the complaints which women bring to the health services are related to some type of violence^(^ 5 ^)^.

Women who experience violence present specific health problems and health needs, such that health practices directed towards them must take as a work object the needs generated by life processes which are common to this social group. It is considered that the work for recognizing and meeting the health needs of women who experience violence must presuppose the denaturalization of inequalities between the sexes and promote women's empowerment. 

One study undertaken in the Municipality of São Paulo, in 19 primary care services, in which 3,193 women were heard, showed that physical and/or sexual violence from an intimate partner in life was experienced by 45.3% of women, such that one in three women using the health services had already suffered violence in her life^(^ 6 ^)^. However, violence remains an invisibilized phenomenon as a demand in these services. One study which investigated the professional practices of the ESF directed towards women who experience violence^(^ 1 ^)^ detected that the professionals recognize the importance of embracing the women who bring this demand, but feel themselves to be impotent and afraid of becoming involved with the situation. This importance is reiterated in various other studies focussing on a similar group when they refer to professional attitudes and practices related to gender violence^(^ 7 ^-^ 8 ^)^_._

In the light of the above, it is argued that in the perspective of a generified practice^(^ 9 ^)^ of health, various possibilities for investigation are found which the universe of the changes in the area of health in the perspective of gender shape. In this study, we emphasize the changes which operate in the ambit of attention to the health needs of women who experience violence in São Paulo. 

The present study aimed to understand the limits and evaluative possibilities of the Family Health Strategy in relation to the recognition of health needs of women who experience violence. 

## Method

This study had a qualitative approach, and was undertaken in a Primary Healthcare Center (UBS) which operates under the Family Health Strategy (ESF), located in the district of Capão Redondo, in São Paulo (SP). This is a region with high rates of violence of all types and poor quality of life. 

The data were collected through in-depth interviews with 22 health professionals who made up the multi-professional teams, and with 13 women who use the service who had experienced situations of gender violence, selected according to different inclusion criteria: among the health professionals, the inclusion was sought of at least one professional from each category of the five teams which made up the UBS which constituted the study scenario, respecting their interest and availability for participating in the study. Among the women using the service, those were invited who, in the period determined for this specific stage of data collection, were waiting for some form of attendance in the waiting room of the health service. Those who showed interested in participating when they learnt about the study had an interview arranged for a later time. The interviews were held by the researcher individually in an attendance room of the health service, were recorded, and were later transcribed and subject to discourse analysis. 

As its theoretical framework, the study adopted the conceptual field of Collective Health, based on the Marxist conception of needs^(^ 10 ^)^ and the role of gender in the determination of the women's health-illness process. For the group presented in the present article, the results were analyzed according to the analytical category of *health needs*.

The study was approved by the Research Ethics Committee of the School of Nursing of the University of São Paulo (Process 822/2009/CEP/EEUSP).

## Results

Based on the analysis of the women's and health professionals' accounts, the emerging themes were identified based on the nuclei of meaning of the text which allowed the organization of the thematic blocks of signification which guide the construction of the empirical categories presented below.

### Gender violence and its relationship with the needs related to the conditions of living in the understanding of the health professionals 

The analysis of the accounts reveals the recognition of needs related to maintenance of life, such as housing, work, food and habits, among others, as the accounts below reveal. 

*Assistance in relation to food and income [...] and managing to find housing *(Professional -4).

*She needs security [...] *(Professional -2).

*First, she needs to stop taking drugs that she uses, and also to improve how she eats *(Professional-1).

*She has addictions that she can't free herself from because she practically lives inside a crack house *(Professional 1). 

*It is a region where there are lots of criminals and she is terrified that he will kill her mother. As a result, she continues to subject herself to this individual [...]. He is an extremely wanted criminal *(Professional-3)*.*

*The family, the situation she lives in, the context of the house, the conditions [...]. Whether she is eating well *(Professional-3).

The family structure was related to the health needs, emphasizing, among the cases, families in which other members experienced domestic violence; women who neglected care for the house and their children; family members with mental disorders; pregnancy in adolescence and family conflicts. 

*There are various situations which generate this violence, it's not only unemployment. It is a lot of things, family breakup: the mother who abandoned the father with various children, the son who is a criminal [...] *(Professional-7).

Some interviewees pointed to meanings which blamed the women as responsible for the violence experienced. 

*The women subject themselves to a lot of things because of liking somebody. This attitude has to be changed, they have to start valuing themselves [...] Only people that want to get hit, they could leave this person *(Professional-13).

On the other hand, some discourses also revealed possibilities for recognizing needs related to the conditions of life which were more linked to social determinants. 

*Social needs [...] because there isn't a serious organic component. It's more a reflection of a sick social question, where the patient ends up losing sleep because she can't manage the little income which she has, etc. *(Professional-20)*.*

### Autonomy as a structuring need for confronting violence 

In some aspects, the discourses indicated the recognition by the health professionals of health needs which have to do with *autonomy*. The meanings raised in the accounts shape these needs related to the woman as the subject of her life, as well as to the need for strengthening to manage the conflicts. 

Also observed was the recognition by some professionals of needs which have to do with conditions which optimize the women's strengthening and autonomy as essential for confronting the violence, as with the example of self-esteem, as well as the need related to social production as a condition for women's autonomy and liberation. 

*The need for managing conflicts, such that they may become a lesson and a challenge, so that she may continue being in charge of her life *(Professional-21).

*The case of financial dependence, trying with the social worker to get other activities so that she can produce, and not be dependent on her husband [...] *(Professional-22).

Accounts from women who experienced violence also revealed social production to be a need intrinsically related to autonomy and to coping with the situation of oppression which characterizes her social reproduction.

*I need a job [...]. At the moment, my solution is to live by his side in the way I am living. And to wait for the baby to be born, to get a job and see what I can do. It's going to be difficult, with a baby *(Service user-11).

### The medicalization of the health needs: the mobilization of the biologicist knowledge as an instrument for health work in the care for the victimized women 

When not reduced to the body, the needs were translated as needs related to mental health, still revealing the fragmented and factorial focus of public health in the view of the health services. 

*The woman's health is good. She's just skinny. Her exam today was all good, there was no anemia, there was nothing. She's always laughing [...] And, if she wants to arrange an appointment every week, who am I to stop her? *(Professional -7).

*She needed to see the psychologist because of the crying [...] and take some sort of medication, she's not sleeping *(Health professional- 5).

The results presented revealed that even when the woman verbalizes the violence suffered to the health professional, this either identifies it in other ways, such as by the physical injuries; as we observed, the professional does not take the problem as a demand of the health field.

*I felt unsupported because of not knowing how to deal with the situation, because the issue is more social than medical, and as we don't have a social service here in the Family Health Program, we are kind of lost, we end up being a stop-gap measure *(Professional 5).

The accounts also revealed the feeling of impotence on the part of the health professionals regarding problems and needs which do not follow the dominant medicalizing logic, as the account below depicts. 

*A feeling of "hey!" I would need other measures for a result, for that person to have a more dignified life [...] I feel as if there is nothing I can do to help in some cases *(Professional-20).

### The relational dimension of the health work: The bond as a possibility for strengthening victimized women 

The relational dimension of the health work was emphasized in the discourses of the need related to listening and to the creation of bonds as a possibility for strengthening women who experienced violence. In this study, a significant proportion of the professionals, as well as of the women who use the service, refer to the need for the women who experienced violence to have somebody in whom they can trust, who can listen to them and embrace them in the health service. 

*Sometimes she thinks that she is ill, she arranges an appointment, she comes here, sometimes she comes here and says that she thinks she has stomach-ache, but actually I think that what she wants is to talk [...] *(Professional-7).

*[...] She always arranged some situation in order to come, I think that it was in order to get out of the house, to have support *(Health professional-10).

*There needs to be a space in which she can exchange her experiences with those of other people *(Professional -21).

In the discourses, the listening and the bond also emerged as health needs which were felt and recognized. 

*This clinic helps me already because, as soon as I leave the house, I am so happy. All I have to do is leave the house to come to the clinic or to get milk for the boy *(Service user- 10).

*What I really need is somebody who understands, I find it difficult to cry, to open up to somebody, it's very hard [cries] *(Service user-7).

In the women's discourses, the overcoming of traumas, respect and happiness were needs recognized as possibilities for strengthening based on embracement, the bond and listening in the health service. Based on this aspect, we understand that, when talking of needs felt, the women emphasize essentially human needs, in the same way as they value the human face of the professional practices when they refer to the health need. 

*What I needed most was for people to respect me and for me to have peace in my life. I pray to God every day; I want to be happy *(Service user-5).

*I needed to get it out of my mind. I want to go back to having a normal life, to being a happy person *(Service user-4).

## Discussion

The analysis of the accounts reveals that the recognition of the needs by the health professionals has to do with the *conditions of life* as determinants of the health-illness process, although they limit their meaning to conditions external to the human being, through a multifactorial understanding of the environment. 

The family structure, recognized by the health professionals as a determinant of the violence, concretizes a large part of the social reproduction, such that the professionals understand its destructured composition as an aspect also related to the determination of the violence and relate it to the conditions of life which are common among the families of the territory covered by the study scenario. One can also observe the responsibilization of the women for this determination arising from a family structure which differs from the ideal, socially-legitimated nuclear family. 

Some interviewees pointed to the determination of the violence centered in the singular dimension, in which the woman, or the situation, is understood as a cause or trigger of the violence, in an acritical and degenerified view of the problem. The meanings revealed in the accounts reveal the conditions of life based in an understanding centered on the individual, in her singular dimension. 

In the professionals' discourse, it is observed that the health needs are related to poor living conditions, unemployment, lack of access to income, housing, food, and drug addiction. The findings strongly show the social conditions of exclusion to which the families who live on the outskirts of the major cities are subjected^*^. 

As a result, needs related and produced in this social structure are possible to capture, even prior to the individual expression which is presented disguised as a health demand in the health service. Through the processes of exhaustion evidenced in the territory, this characteristic must be the basis of the entire organization of the practices, so as to overcome the current model of medicalized, fragmented and individualistic care. 

The discourses have to do with personal self-management, individual internal motivation, and decision-making as needs which are related to *autonomy *for the emancipation from oppression of women. The risk of reductionism, however, may be implicit in this notion, which translates into the de-responsibilization of the service in relation to the problem. 

The meanings raised in the accounts shape the autonomy as a need related to the woman as the subject of her life, as well as to the need for strengthening for the management of the conflicts. In various accounts, autonomy appeared related to the conquest of financial independence and/or work in the public world, and was revealed as a need for women for their transformation and liberation from oppression and violence. This was a convergent aspect with the professionals' discourses. In revealing a discourse which points to autonomy, the interviewees overcome the understanding of needs for maintaining life, pointing towards essentially human needs, which means an object theme which can broaden, indicating a quality which is revealed in the discourses, although within the limits mentioned above. 

Studies demonstrate that the women who experience violence have something more to say besides the complaints which they bring to the health services, that is to say, in the gender relationships, the way male domination tries to impose silence on the women involved in violent relationships, such that, when these seek the health services, they bring an indirect discourse and nearly always speak of other complaints^(^ 4 ^,^ 8 ^)^. This aspect can also be explained by the responses which the women, historically, have received from the health services, such that, when their needs are translated into demands for treatment of health problems already in place, the services bring these needs about in the women. Thus, they themselves translated these into physical demands with the aim of being embraced by the service. 

The health needs brought by the women were recognized by the professionals as demands related to the needs for care of physical health issues, which we translate into what can be termed the *medicalization* of the health needs. The accounts point to the recognition of health needs reduced to the women's reproductive health, at an individual level. The mention of the need for specialized attendance in mental health was significant, revealing what the studies have also indicated: the mind-body dichotomy in the health work^(^ 3 ^,^ 8 ^,^ 11 ^)^ **.** In this way, when a problem is not inscribed on the body or does not follow the medicalized logic, as happens with violence, it is inscribed on the mind and requires specific attention in the area of mental health. 

The medicalization revealed in the accounts can be translated into a greater prescription of analgesics, tranquilizers and referrals to the mental health services for women who live in situations of violence, although the diagnosis may not be recorded. This way, as the root of the problem is not examined, conduct can end up strengthening destructive processes, as well as not helping in confronting the problem^(^ 3 ^)^.

Medicalization, identified in the professionals' discourses, when they spoke of the *recognized* needs, was not convergent with the women's discourses when they spoke about the *felt* needs. Therefore, the results presented reveal that even when the woman verbalizes the violence suffered to the health professional, or when this identifies her in other ways, such as from the physical injuries, the professional does not take the problem as a demand of the health field. 

The discourses have to do with the meanings which relate social problems as part of the specific services of this area, understanding that, when she acts in these aspects, the health professional is filling a gap which is not part of her role. Hence, thinking about the health needs of women who experience violence as isolated needs in the individuals and families and, furthermore, as a social problem whose attention falls to other fields and not that of health, is to remain in an abstract situation which does not consider, and does not intervene in, the confronting of the determinants of health. 

Collective Health constitutes the theoretical-practical field which articulates the health sciences with the social sciences, being an area which is concerned with the social aspects related to the health-illness process, which interact, dialectically, with the biological, psychological and subjective aspects. It questions the reductionism which naturalizes the social and reproduces the medicalizing interpretation of illness and of the actions in health, interpreting and acting on the determinants of health in the singular (individual), particular (of the groups) and structural (of the political and ideological structure of the broader society) dimensions of the reality.

Diverging from this interpretive picture, the medicalizing reality, captured in the discourses presented here, reproduces the instrumental, biologicist and multifactorial knowledge, centered on the individual and the illness. In addition, the violence appears within a degenerified and naturalized understanding. This reality results from an entire historical process of construction of healthcare in our society and, as a consequence, of academic training which remains in the biomedical and androcentric mold. 

Even when enunciated, domestic violence is not recognized, neither is it included in the definition of needs by the health professionals for the work, as there are no technical actions foreseen for this^(^ 4 ^)^. If, on the one hand, the women who experience violence seek the health services with demands relating to other complaints, on the other, this represents a challenge to be achieved by the health services: the translation of these dimensions into the need to produce them, considering violence and the subordinateness of gender as determinants of this process. These findings reinforce the importance of the development of measures for the recognition of the violence and of the needs which it produces in the various instances of care for the women. The professionals' reports reveal the impotency felt when they face violence as a problem which is not within the field of action based in the medicalizing logic of health. 

In this study, the interface between the ESF health professionals' discourse and women victimized by gender violence, regarding the recognition of health needs, reveals the needs related to autonomy, to the bond and to listening as common aspects in the discourses of the health professionals and the victimized women. This aspect points to the essentially human needs in the intersection between the meanings which the health needs take on for the two groups of subjects. In relation to the victimized women, the recognition of the essentially human needs (peace, overcoming traumas, happiness, life without violence) was predominant. The medicalized needs, those related to the conditions of life and the maintenance of life, revealed in the health professionals' discourses, were not identified in the discourses of the users of the service. 

Based on the *bonds* established between professionals and service users, determined by the continuous attendance and by the proximity which the ESF allows, raise possibilities for the capturing of violence and of the needs. Furthermore, in the dialogue and in the listening established in this relationship, there is the potential for the overcoming of traumas and for the strengthening of the women. We speak of potential as it does not constitute a concrete logic operationalized in its entirety, still constituting a possibility in the objective reality of the health services^(^ 4 ^)^.

It is important that the listening established in the contact between professionals and service users should not be translated in practice to a simple dialogue permeating the care. Qualified listening presupposes comprehensive care, an attentive view which, in the care for women who experience violence, must be mediated by a generified instrumental knowledge, which covers the service users' needs, recognized as subjects of their existence, and inserted in a society which determines the subordinateness and violence.

## Final considerations

The discourses brought by the victimized women and by the health professionals reveal divergences and convergences, revealing the essentially human needs as the interface of the meanings which the health needs take on for these subjects. The needs which have to do with female autonomy and with the bond in the relationship between professionals and women who use the service are revealed as common aspects in the discourses. However, what is said between the lines of the accounts reveals a gap in the use of the strengths of this interface by the health professionals.

The women refer to the essentially human needs not referred to by the professionals. Besides this, the recognition of the needs for good conditions of life and medicalized needs translated into physical health issues by the professionals were not significant aspects in the discourses of the women who used the service. Regarding the conditions of life, in particular in the territory permeated by social exclusion and violence, the women's non-recognition of the relationship between the condition of life and health needs may be related to the absence of criticism regarding the relationship between health and society, reflecting an idealized vision which results from the historical construction of the female in society. 

The medicalization of health needs, as well as the predominance of existential needs in relation to the essentially human needs reveals a mismatch between the needs of the victimized women and their understanding by the professionals. 

Thus, this study reveals divergences, pointing to limits which must be considered in the planning of effective health care for this specific social group, and possibilities which must be deepened and strengthened as potential for the prevention and confronting of gender violence in the ambit of health based on the re-reading of the needs of the victimized women. 

The need is urgent for recognition of the violence as a problem and a demand whose attention is inherent to the health services. The translation of the demands brought by the women in the needs which produced it represents a challenge to be achieved. The recognition of these needs also presupposes the consideration of gender violence and subordinateness as factors which generate this process. Thus, the work which qualifies the healthcare of the women in situations of violence must overcome the biomedical model of care, limited to the process of biological reproduction, which still characterizes the majority of the work processes of the practices in women's health, remaining faithful to the positivist conception of science. The overcoming of this model entails reviewing professional practice, given that, in the perspective of the emancipation of women from oppression, it is through the critical knowledge regarding health needs as a consequence of the situation of oppression that the gender approach closes one of the instruments which must guide the entirety of the work of the professional practices in this area.
